# Supplementary material for: Predicting treatment benefit in multiple myeloma through simulation of alternative treatment effects
Source: Nat Commun. 2018 Jul 27;9:2943. doi: 10.1038/s41467-018-05348-5 (PMC6063966; doi:10.1038/s41467-018-05348-5)
Supplement: Supplementary file 1 — Supplementary Information [file 41467_2018_5348_MOESM1_ESM.pdf]

Predicting treatment benefit in Multiple Myeloma through simulation of alternative treatment effects

Ubels et al.

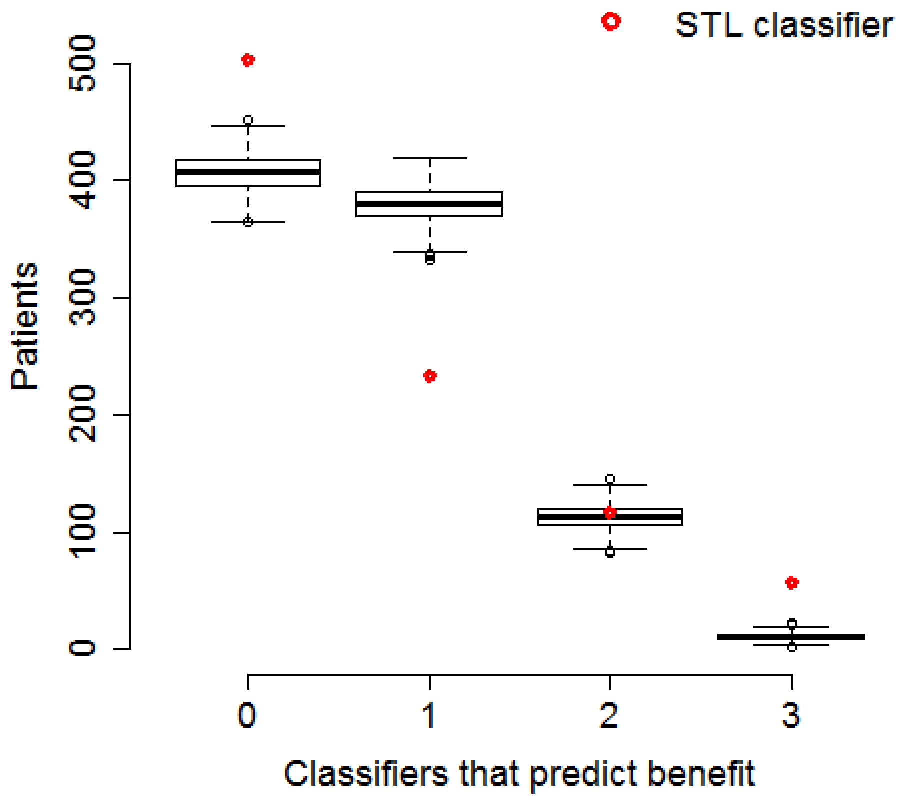

Supplementary Figure 1. We computed for how many patients the three classifiers trained in the different folds of the cross validation agree on class assignment. The values on the x-axis represent the number of classifiers that classified a patient as benefitting from treatment. A value of 0 means that all three classifiers classified a patient as 'no benefit' and the value of 3 (which is the maximum) means all classifiers agreed on the assignment to class 'benefit'. These are the red dots in the plot. We also generated 10 000 random labelings per training fold, with the same proportion of patients labeled 'benefit' and 'no benefit' as in the labelings found by STL to obtain a background distribution of the expected overlap by random chance (boxplot). Since the number of patients for which all three STL classifier agree (i.e. the patients with either a value of 0 or 3) is larger than expected by random chance, the concordance between the STL classifiers is significant.

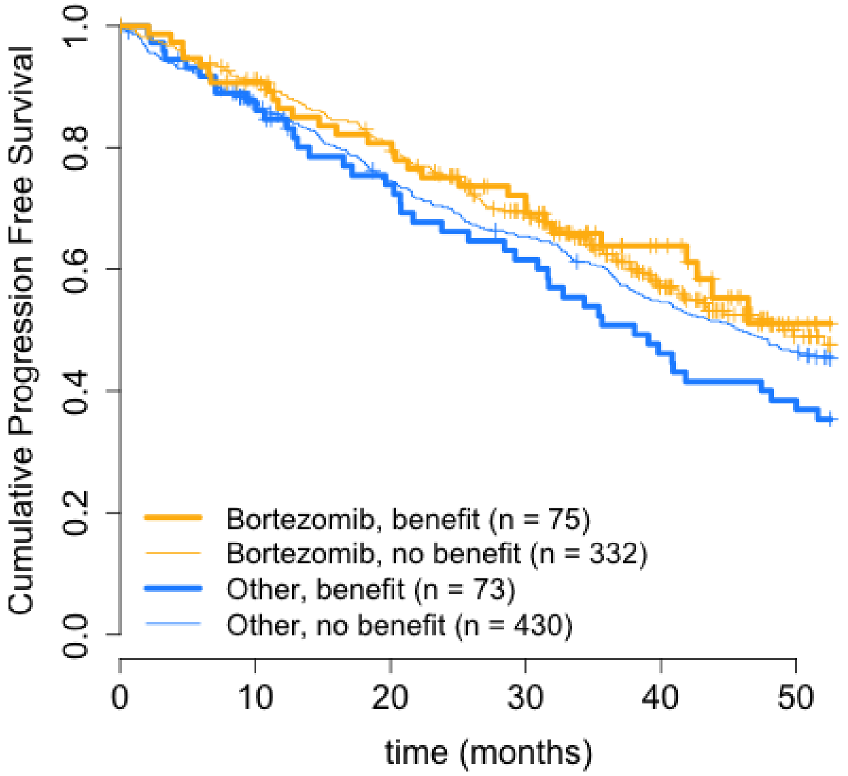

HR ‘benefit’ class = 0.56, p = 0.02

HR ‘no benefit’ class = 0.77, p = 0.02

Supplementary Figure 2. Kaplan Meier of the classification of the bortezomib dataset using random gene sets. In the class ‘benefit’ an HR of 0.56 (95% CI 0.34 – 0.90, p = 0.02, n = 148) is found and in the class ‘no benefit’ an HR of 0.77 (95% CI 0.62 – 0.96, p = 0.02, n = 762).

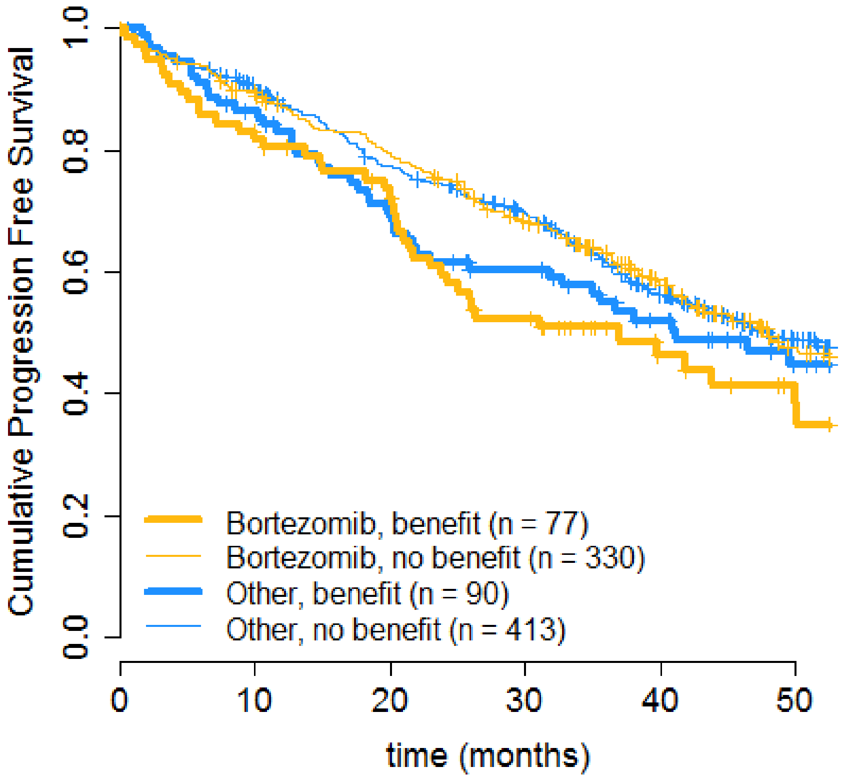

HR ‘benefit’ class = 1.09, p = 0.69

HR ‘no benefit’ class = 0.95, p = 0.65

Supplementary Figure 3. Kaplan Meier showing the survival curves in validation when the treatment labels are shuffled, i.e. patients are in silico randomly assigned to the either the bortezomib or no bortezomib arm. An HR of 1.09 (95% CI 0.71 – 1.67, p = 0.69, n = 167) in the class ‘benefit’ and an HR of 0.95 (95% CI 0.77 – 1.18, p = 0.65, n = 743) in the class ‘no benefit’ is observed. It is expected that no performance is observed, since the relationship between the gene expression data and the treatment specific survival is destroyed.

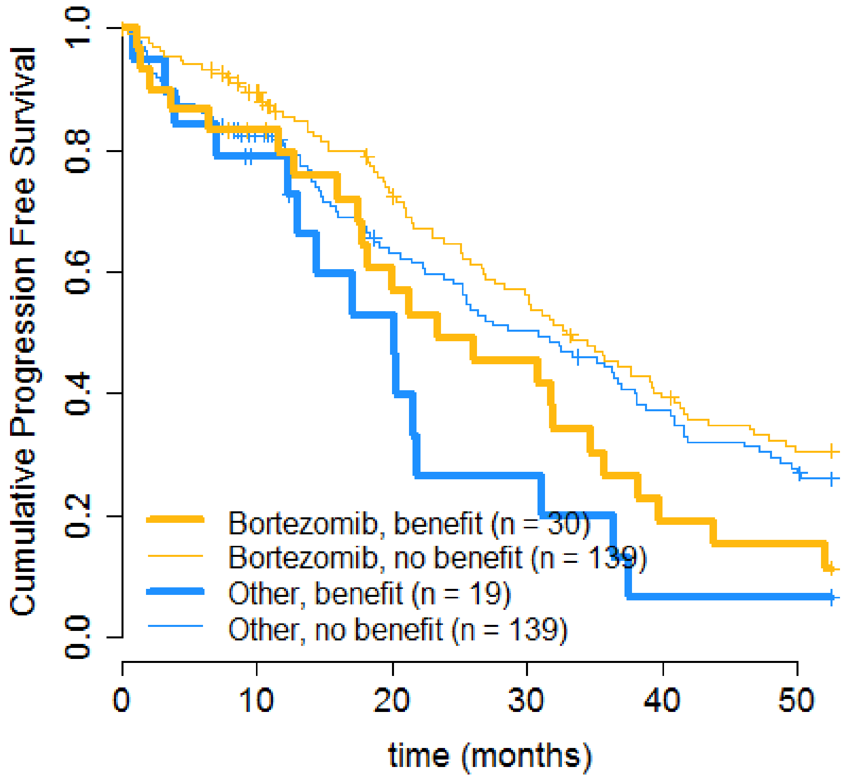

HR ‘benefit’ class = 0.69, p = 0.26

HR ‘no benefit’ class = 0.85, p = 0.27

Supplementary Figure 4. Validation performance of the STL classifier in the H65 dataset when the classifier is trained on the combined TT/H65 dataset. An HR of 0.69 (95% CI 0.36 – 1.32, p = 0.26, n = 49) is observed in class ‘benefit’ and an HR of 0.85 (95% CI 0.63 – 1.14, p = 0.27, n = 278).

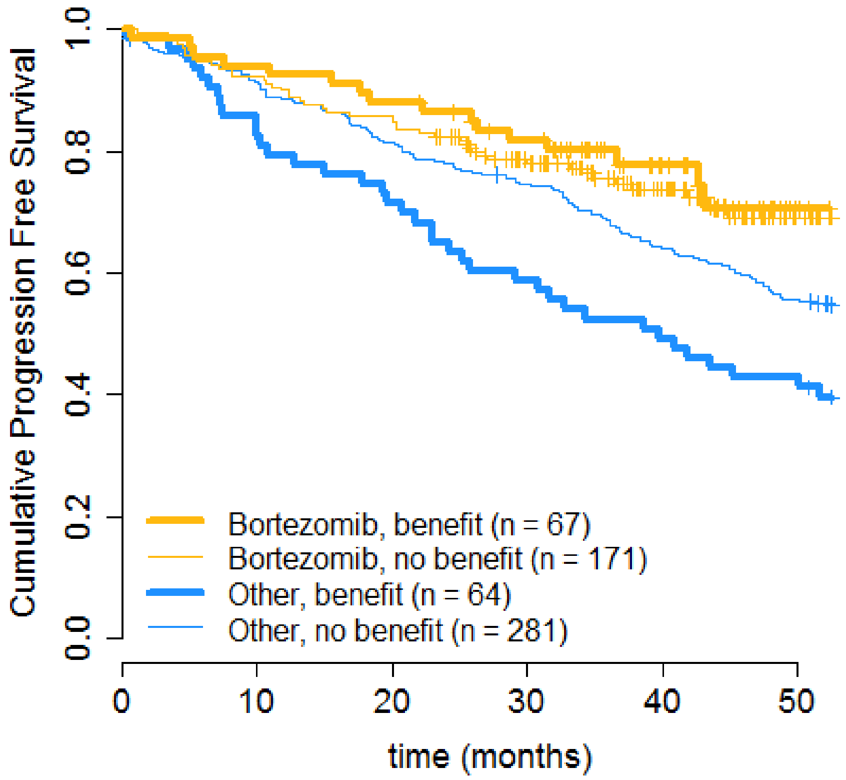

HR ‘benefit’ class = 0.38, p = 0.002

HR ‘no benefit’ class = 0.71, p = 0.05

Supplementary Figure 5. Validation performance of the STL classifier in the Total Therapy dataset when the classifier is trained on the combined TT/H65 dataset. An HR of 0.38 (95% CI 0.21 – 0.69, p = 0.002, n = 131) is observed in class ‘benefit’ and an HR of 0.71 (95% CI 0.50 – 1.00, p = 0.05, n = 452) in class ‘no benefit’.

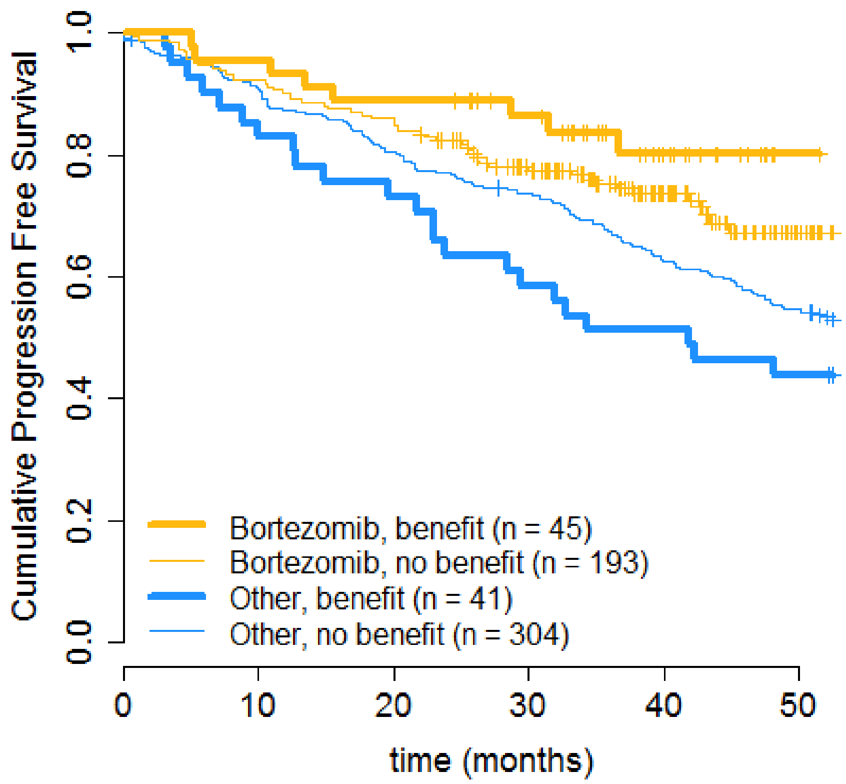

HR ‘benefit’ class = 0.28, p = 0.00098

HR ‘no benefit’ class = 0.71, p = 0.038

Supplementary Figure 6. Kaplan Meier showing the survival curves when the STL classifier is trained within the Total Therapy (TT) datasets, excluding the data from the HOVON65 (H65) trial. An HR of 0.28 (95% CI 0.13 – 0.60, p = 0.00098, n = 86) is observed in class ‘benefit’ and an HR of 0.71 (95% CI 0.51 – 0.98, p = 0.038, n = 497) is class ‘no benefit’. The HR found in class ‘benefit’ is far lower than the HR found in validation when TT and H65 are combined.

Supplementary Table 1. An X indicates a patient included in the study (rows) received that drug (columns).

	**bortezomib**	**doxorubicin**	**dexamethasone**	**thalidomide**	**cyclophosphamide**	**cisplatin**	**etoposide**	**vincristine**
H65 - PAD arm	X	X	X					
TT3	X	X	X	X	X	X	X	
H65 - VAD arm		X	X	X				X
TT2		X	X	X	X	X	X	X

Supplementary Table 2. Performance of nearest mean classifier when different percentages are used to define class 'benefit'

	5%	10%	15%	20%	25%	30%	35%	40%	45%	50%
HR	1.05	1.02	0.72	0.85	0.96	0.65	0.64	0.74	0.75	0.65
p-value	0.85	0.96	0.26	0.53	0.86	0.08	0.09	0.23	0.23	0.08
Size class 'benefit'	0.30	0.20	0.27	0.35	0.36	0.41	0.42	0.44	0.46	0.49
Mean accuracy	0.41	0.50	0.49	0.49	0.58	0.56	0.56	0.55	0.55	0.56

Supplementary Table 3. Performance of random forest classifier when different percentages are used to define class 'benefit'

	5%	10%	15%	20%	25%	30%	35%	40%	45%	50%
HR	0.61	0.56	0.90	0.91	0.91	0.75	0.84	0.74	0.80	0.75
p-value	0.41	0.23	0.73	0.80	0.73	0.29	0.51	0.23	0.36	0.24
size class 'benefit'	0.11	0.13	0.20	0.16	0.25	0.30	0.37	0.41	0.44	0.50
accuracy	0.70	0.69	0.70	0.69	0.69	0.68	0.68	0.68	0.67	0.65

Supplementary Table 4. Performance of support vector machine when different percentages are used to define class 'benefit'. When using 5% no patients were assigned to class 'benefit' in validation, making it impossible to compute an HR.

	5%	10%	15%	20%	25%	30%	35%	40%	45%	50%
HR	NA	9.47E +08	0.41	0.96	0.81	1.01	0.85	1.13	0.83	0.70
p-value	NA	1.000	0.53	0.95	0.67	0.97	0.57	0.66	0.46	0.16
size class 'benefit'	NA	0.013	0.02	0.05	0.10	0.23	0.30	0.34	0.40	0.50
accuracy	0.98	0.963	0.93	0.92	0.81	0.78	0.72	0.71	0.79	0.71

Supplementary Note 1

The parameters k and γ determine the classification boundary. For this reason, they are optimized using an exhaustive grid search which chooses the optimal combination. To investigate how sensitive this optimization is, we investigated how small changes to the parameters affect the HR found in validation. In essence, a smaller γ leads to a smaller class benefit. We show the effect of changing the γ parameter in two scenarios: leaving all other parameters as is (Supplementary Figure 7) and when also retraining the threshold T which determines how many classifiers need to agree on the 'benefit' classification (Supplementary figure 8). The classifier is robust to (small) changes in these parameters, which is a desirable feature of a robust classifier. As can be seen in Supplementary Figure 7, when γ decreases, the HR also decreases since a smaller class benefit is identified. This is consistent with our observation that a smaller class benefit leads to a lower HR (Figure 2c). When threshold T is also reoptimized, the HR stays relatively constant when γ is changed, since the threshold T is chosen so at least 20% of the patients are classified as class 'benefit'. Supplementary Figure 9 shows the number of patients who receive a different class assignment when γ is changed, again without reoptimizing threshold T (black line) and with reoptimization (red line). When threshold T is reoptimized, few patients change classification, showing different settings for γ would identify the same patients as benefitting from bortezomib. We also investigated how sensitive the classifier is to changing the number of genesets in the classifier (with reoptimization of threshold T, Supplementary figure 10). The red line indicates the validation HR we originally found. As can be seen, there are many settings which achieve a similar or better validation performance, indicating the classifier is also not very sensitive to the exact number of gene sets included.

We also investigated the training HR found for all k and γ combinations of three of our top- performing genesets (Supplementary Figures 11 - 13). Note that these gene sets were the best performing in one of the folds and are not necessarily overrepresented in the final classifier. The y-axis show the different settings for k and the x-axis the different settings for γ. A yellow color indicates a low HR, a blue color a high HR and white indicates too few or too many patients were included in class 'benefit' when this combination was used. What can be seen is that a low setting for k (meaning few prototypes) leads to the most favorable HRs. Also here can be seen that small changes in k and γ do not lead to large changes in HR.

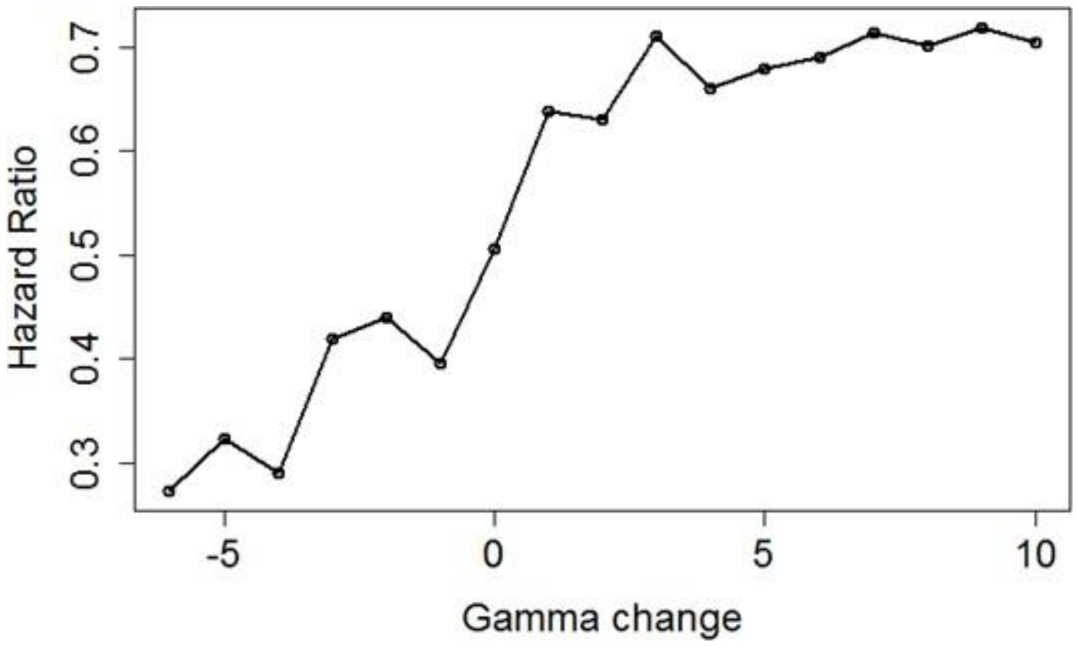

Supplementary Figure 7. The effect of changing γ on the HR when threshold T is not re-optimized. The y-axis shows the validation HR and the x-axis the change in γ.

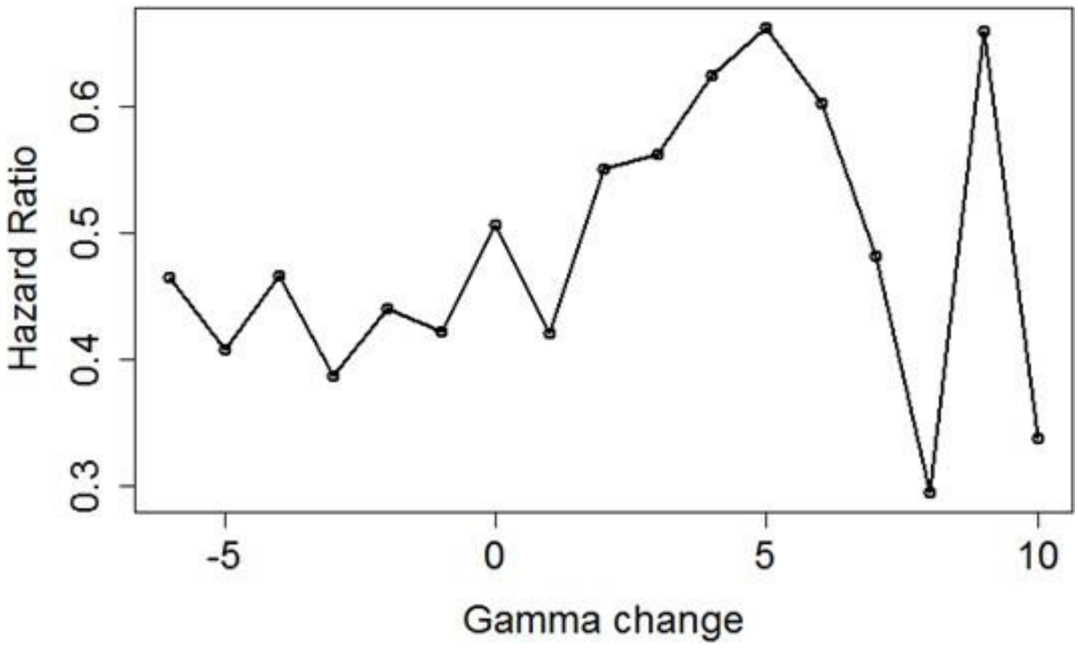

Supplementary Figure 8. The effect of changing γ on the HR when threshold T is re-optimized. The y-axis shows the validation HR and the x-axis the change in γ.

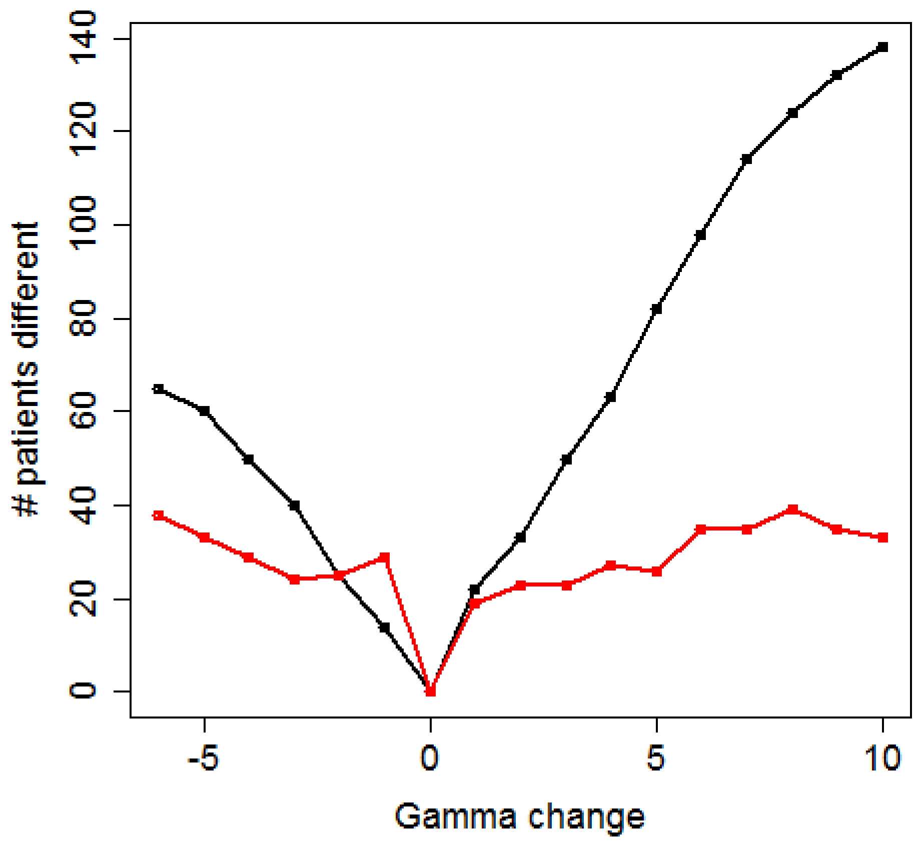

Supplementary Figure 9. The number of patients who change from class 'benefit' to class 'no benefit' or vice versa when γ is changed. The red line shows the difference when we re-optimize the threshold T, the black line when we do not.

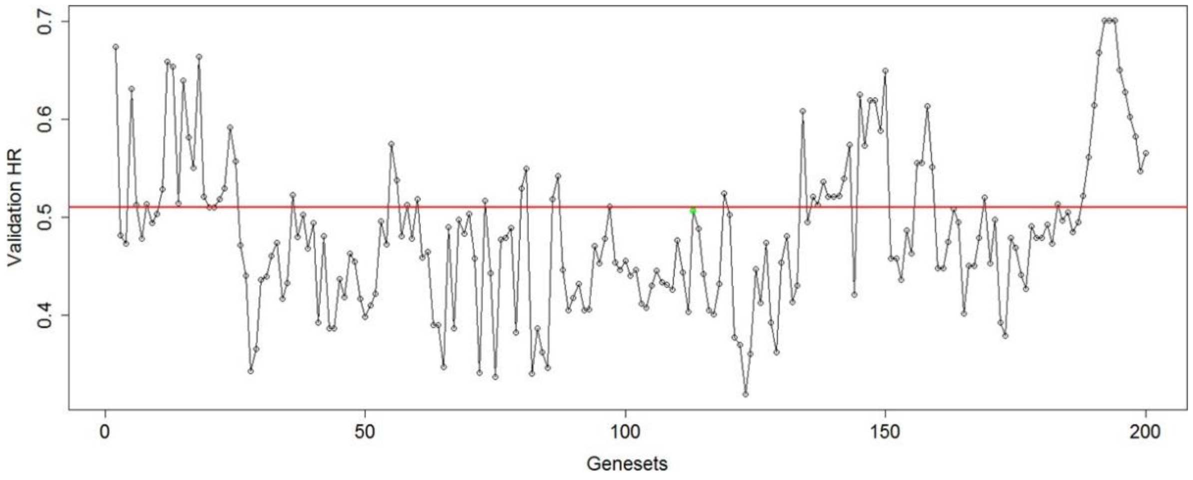

Supplementary Figure 10. The validation HR found when a different number of genesets is included in the final classifier. The red line indicates the validation HR found with the original classifier.

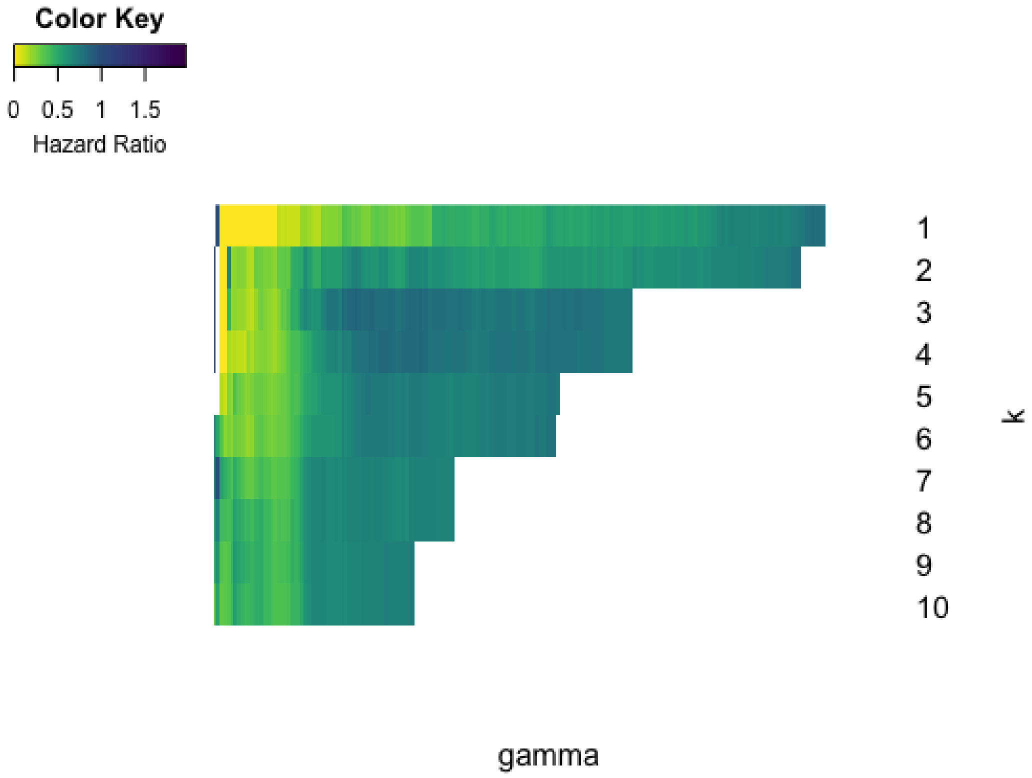

Supplementary Figure 11. Training performance for different combination of k and γ, using GO category olfactory bulb axon guidance. The y-axis show the different settings for k and the x-axis the different settings for γ. A yellow color indicates a low HR, a blue color a high HR and white indicates too few or too many patients were included in class 'benefit' when this combination was used.

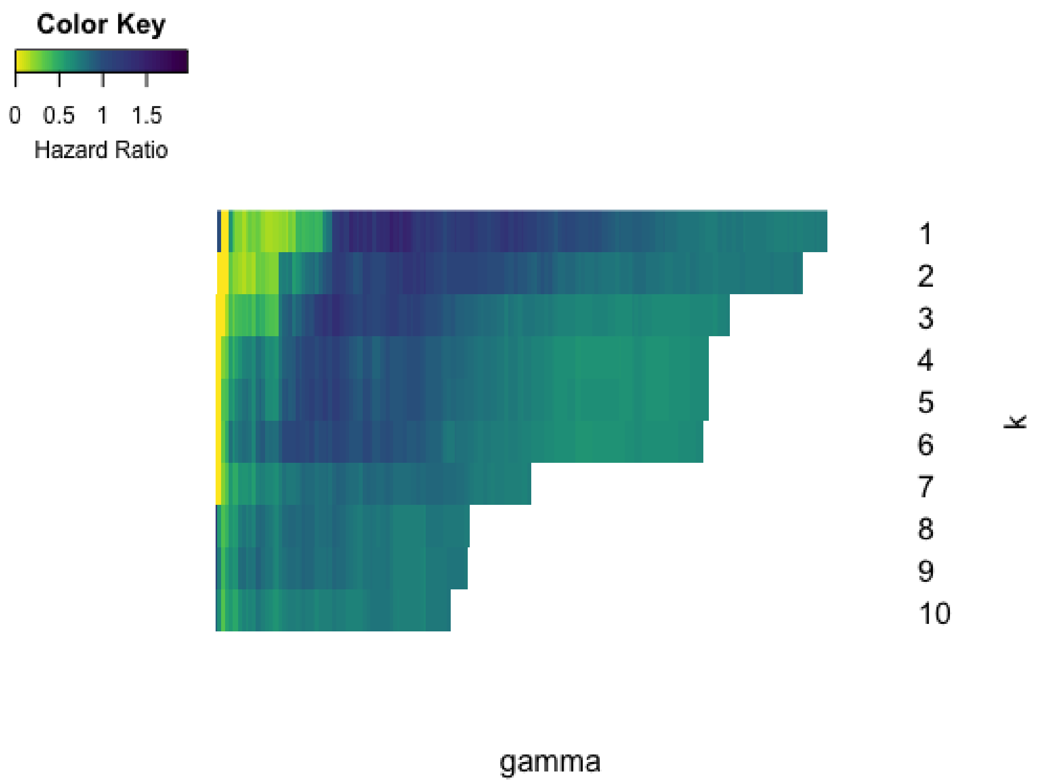

Supplementary Figure 12. Training performance for different combination of k and γ, using GO category peptidoglycan receptor activity. The y-axis show the different settings for k and the x-axis the different settings for γ. A yellow color indicates a low HR, a blue color a high HR and white indicates too few or too many patients were included in class 'benefit' when this combination was used.

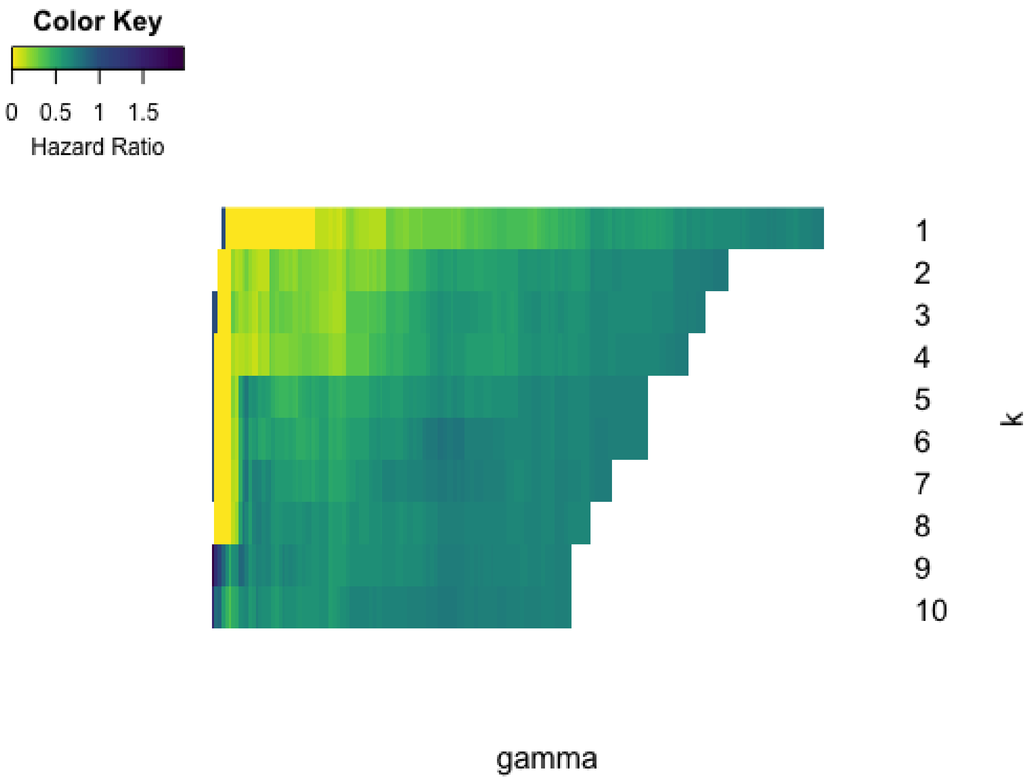

Supplementary Figure 13. Training performance for different combination of k and γ, using GO category IgG binding. The y-axis show the different settings for k and the x-axis the different settings for γ. A yellow color indicates a low HR, a blue color a high HR and white indicates too few or too many patients were included.
